# Hybridized nanogenerators for effectively scavenging mechanical and solar energies

**DOI:** 10.1016/j.isci.2021.102415

**Published:** 2021-04-10

**Authors:** Xue Zhao, Chunlong Li, Yuanhao Wang, Wei Han, Ya Yang

**Affiliations:** 1 Sino-Russian International Joint Laboratory for Clean Energy and Energy Conversion Technology, College of Physics, International Center of Future Science, Jilin University, Changchun 130012, P.R. China; 2 CAS Center for Excellence in Nanoscience, Beijing Key Laboratory of Micro-nano Energy and Sensor, Beijing Institute of Nanoenergy and Nanosystems, Chinese Academy of Sciences, Beijing 101400, P. R. China; 3 Global Energy Interconnection Research Institute Co., Ltd., Nanjing 210000, P. R. China; 4 SUSTech Engineering Innovation Center, School of Environmental Science and Engineering, Southern University of Science and Technology, Shenzhen, Guangdong 518055, P. R. China; 5 Electric Power Intelligent Sensing Technology and Application State Grid Corporation Joint Laboratory, Beijing 102209, P. R. China; 6 School of Nanoscience and Technology, University of Chinese Academy of Sciences, Beijing, 100049, P. R. China; 7 Center on Nanoenergy Research, School of Physical Science and Technology, Guangxi University, Naning, Guangxi 530004, P.R. China

**Keywords:** Devices, Energy Resources, Energy Systems

## Abstract

Solar and wind energy harvesting technology is increasingly an economical and efficient energy form and receives excellent support from government policies worldwide. Various functional and structural nanogenerators based on multi-effects named hybridized nanogenerators have been reported separately or simultaneously to effectively generate the wasted mechanical and solar energy in our daily life. We review the development of hybridized nanogenerators, including the working mechanism of solar and mechanical energies. Moreover, the classification of nanogenerators for scavenging mechanical and solar energies is discussed. The potential applications of hybridized nanogenerators are reviewed. Finally, the challenge and prospective of hybridized nanogenerators and the future explored improvements of output performance, stability, preparation, large-scale utilizing, and efficiency are discussed. The hybridized nanogenerator as the energy technology will be popularized in energy and self-powered sensor systems.

## Graphical abstract

**Figure:**
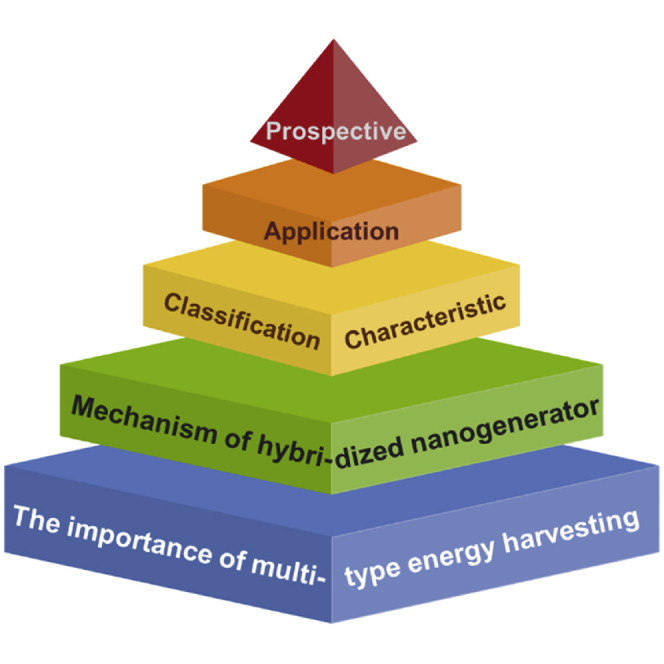

## Introduction

**Figure 1:**
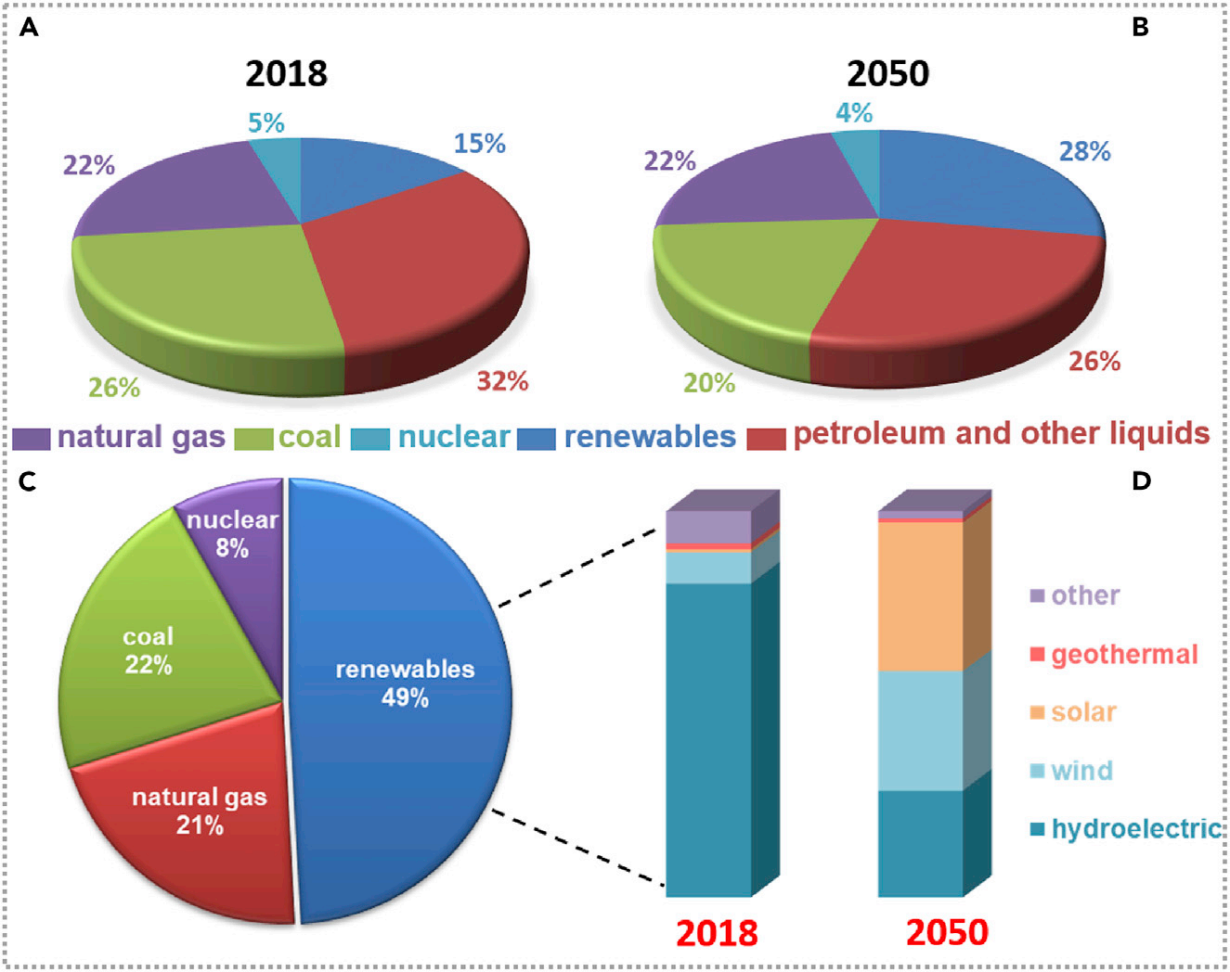
The consumption of different types of energy all over the world (A and B) Primary energy consumptions shared by all forms of energy sources around the world in 2018 and 2050. (C) The source of global electricity generation from renewable energy to 2050. (D) The share of electric energy production from renewables worldwide, among renewable energy sources in 2018 and 2050.

Renewable energy is an inexhaustible, widespread, and environmental-friendly energy type, including solar energy, hydro energy, wind energy, biomass energy, wave energy, tidal energy, geothermal energy, etc. In response to the global energy and environmental crisis, developing a promising energy-technology-based renewable energy is a significant challenge. Figures 1A and 1B exhibit the share of primary energy consumption by energy source worldwide in 2018 and 2050, and all forms of energy consumption are increasing over time. The reference data of the "International Energy Outlook 2019 with projections to 2050″ from the website "https://www.eia.gov/ieo." From 2018 to 2050, renewable energy consumption has been increasing at 3% per year. By the end of 2050, driven by the expansion of electricity demand, global development, and globalization policies, sustainable energy will become a significant source of primary energy consumption. In reference, serving as the fastest-growing source of electricity generation, the utilization amount of renewable energy grew by an average annual rate of 3.6% during the 2018 to 2050 period, and by 2050 the 49% of global electricity will be provided by renewables, as shown in Figure 1C. Figure 1D is the share of global renewable energy generation. Among diverse renewable energy sources, the hydroelectric amount decreased sharply. However, from 2018 to 2050, wind and solar energy contributed the most to power generation, accounting for more than 70% of the total power generation. Solar and wind energy harvesting technology is increasingly an economical and efficient energy form and receives excellent support from government policies worldwide. More and more researchers pay attention to constructing a promising efficient energy-generating technology to eliminate universal solar and wind energy and convert them directly into electricity, which could mitigate global warming and the energy crisis.

Electromagnetic induction is a phenomenon that induced electromotive force caused by changing the magnetic flux through the closed conductive wire. The discovery of electromagnetic induction represents one of the most significant achievements in electromagnetism, which reveals the interrelation between electricity and magnetism's phenomena and brings excellent industrial and technological revolution. Basing upon the Faraday's law of electromagnetic induction, electromagnetic generator (EMG) makes it possible for the mass production and long-distance transmission of electric energy. With the development of technology and alleviating the global energy crisis, EMG gradually plays a crucial role in accelerating the development of social productivity and science and technology, especially being served as a mechanical energy generator. Besides, triboelectric nanogenerators (TENG) (Shao et al., 2020; Wang et al., 2015a; Wang, 2014) were invented in 2012, tactfully designed to scavenge various mechanical energy and generate electric power directly based on a combination of triboelectrification and electrostatic induction.

Similarly, piezoelectric nanogenerator (PENG) based on the piezoelectric effect could be employed to scavenge multiple mechanical energy into electrical energy (Qin et al., 2008a; Xu et al., 2010). Given the above, different types of TENGs, EMG, and PENGs with varying structures could scavenge the easily overlooked mechanical energy in the environment, such as the wind, flowing water, vibrating wave, human movements, and rotating plate. In addition to mechanical energy, widely available solar energy is equally a promising energy form and has a wide range of applications. Solar-energy-generating technology based on photovoltaic effect or photothermal effect possesses characteristic of eco-friendly, simple construction. It could be utilized on a large scale, which has caught more and more attention, and kinds of efficient photovoltaic devices with excellent performance have been constructed.

In this decade, researchers have fabricated multifarious nanogenerators based on triboelectric, electromagnetic, piezoelectric, photovoltaic, and photothermal effects to effectively generate the wasted mechanical and solar energy in our daily life. Various nanogenerators based on hybridized nanogenerators came into existence to collect different renewable forms and could be utilized as energy generators under different environments. With the development of energy-generating technology, multifarious functional and structural hybridized nanogenerators were constructed, which could scavenge considerable energy separately or simultaneously with superior performance compared with single-effect nanogenerators. We review the development of hybridized nanogenerators harvesting mechanical and solar energy, the working mechanism of solar and mechanical energy scavenging. Moreover, the classification of mechanical harvesting energy and solar energy nanogenerator is also discussed according to the forms of mechanical energy and solar cell types, respectively. The diverse application of hybridized nanogenerators is being detailed reviewed. Finally, challenges and prospects of hybridized nanogenerators and the future explored improvement in output performance, stability, preparation, large-scale utilizing, and efficiency are discussed.

## The principle of hybridized nanogenerators

Mechanical and solar energies are concomitant in our environment in most situations; it is necessary to construct hybridized nanogenerators to scavenge various types of renewable energy separately or simultaneously and convert them into electricity efficiently. The hybridized nanogenerator integrating the TENG, PENG, EMG, and different solar cell types, the hybridized nanogenerator mechanism, will be discussed below according to various energy forms.

### The principle of harvesting mechanical energy

To meet the growing demand for electrical energy, environmentally friendly energy technology, such as EMG, TENG, and PENG, has attracted the attention of researchers for their satisfactory ability to scavenge a mass of wasted mechanical energy in the environment, such as the wind, flowing droplets, wave vibration, rotating tire, and human motion in the environment. Various kinds of nanogenerators were demonstrated and served as an energy source for electronics devices or sensors in human civilization. Here we have overviewed and summarized the fundamentals of EMG and TENG.

#### The principle of hybridized EMG-TENG

Triboelectrification is a ubiquitous phenomenon, which can occur between any two dissimilar materials around us. It has been widely known ever since the ancient Greek era. TENG is tactfully designed to scavenge the mechanical energy and generate electricity directly based on triboelectrification and the electrostatic induction effect. The TENG has four foundational working patterns, including vertical contact-separation mode, lateral sliding mode, single-electrode mode, and freestanding triboelectric-layer mode (Shao et al., 2020; Wang et al., 2015a; Wang, 2014). The TENG based on the vertical contact-separation mode has been widely investigated and adopted for its advantages of simple structure and convenient assembly (Chen et al., 2018; Zhang et al., 2015; Zhao et al., 2019). EMG is another standard and efficient technology for mechanical energy scavenging, which is based on the Faraday electromagnetic induction effect. To scavenge and utilize the mechanical energy in the environment more efficiently, the EMG and TNNG are usually integrated into a whole device. The two parts work independently and could be activated either under low-frequency or under high-frequency mechanical excitation (Gao et al., 2020; Quan et al., 2015a, 2015b, 2016; Quan and Yang, 2016a; Wang et al., 2015b, 2016c; Wang and Yang, 2017; Wu et al., 2015; Zhang et al., 2014, 2016c; Zhang and Yang, 2016; Zhong et al., 2015).

**Figure 2:**
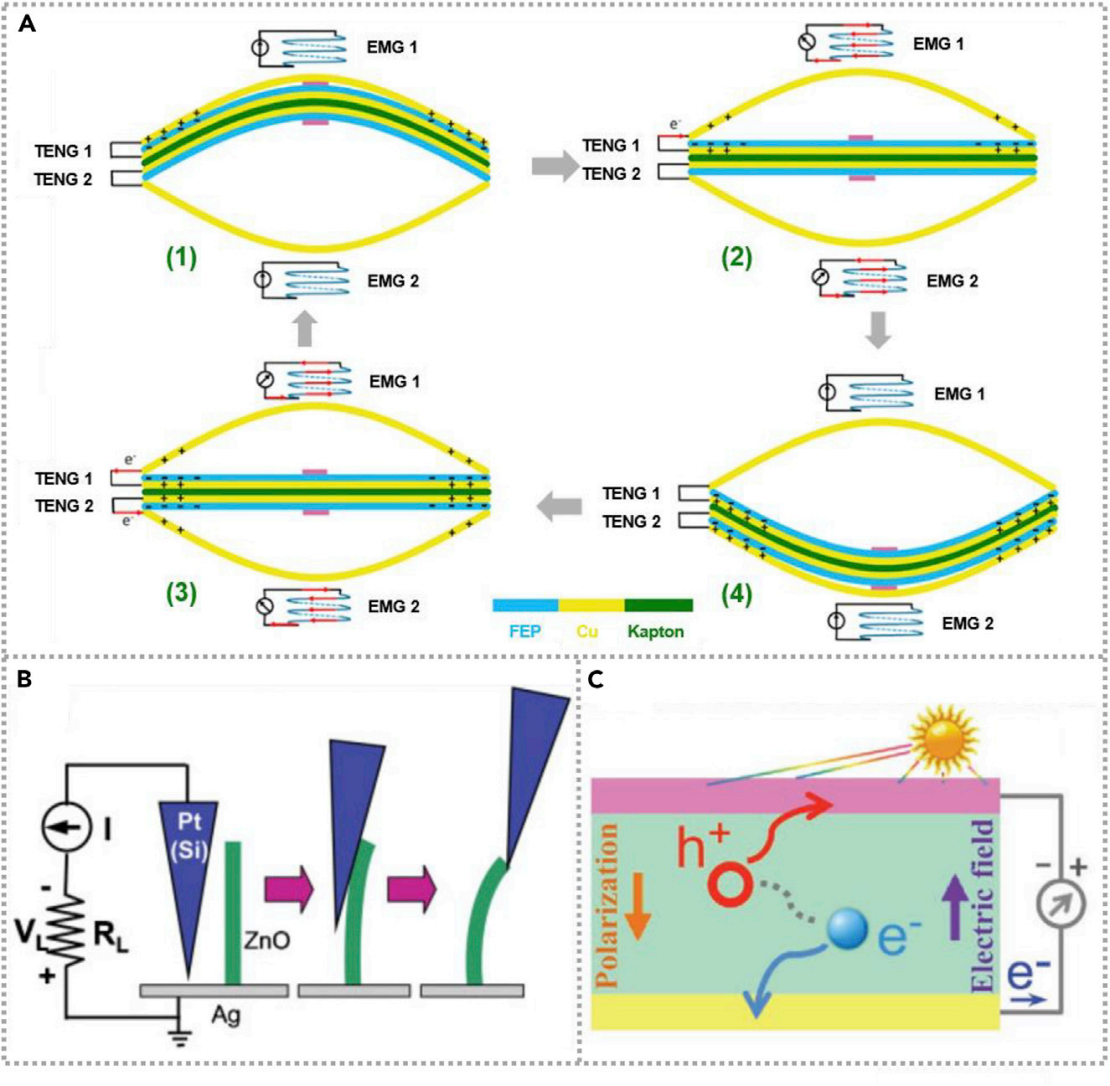
The principle of harvesting mechanical and solar energies (A) Schematic diagrams of the working principle for the hybridized electromagnetic-triboelectric nanogenerator under different conditions. Reproduced with permission, from ref (Wang and Yang, 2017), Copyright 2016, Elsevier. (B) The principle of piezoelectric-nanogenerator-based ZnO nanosheet. Reproduced with permission, from ref (Wang, 2008), Copyright 2008, Wiley-VCH. (C) The schematic illustration mechanism of the photovoltaic effect. Reproduced with permission, from ref (Zhang et al., 2017), Copyright 2017, Wiley-VCH.

A detailed operating principle of a hybridized EMG-TENG is shown in Figure 2A (Wang and Yang, 2017), which demonstrated nanogenerator consisted of two TENGs and two EMGs. FEP films and Cu foils are regarded as triboelectric pairs of TENGs with coils and magnets to make up EMGs. At the initial state, Cu electrodes and vibration film were nicely separated by air gaps, and there was no charge on the surface of both materials. When the wind caused the contact between FEP and the top of Cu, the electrons could transfer from Cu (named as positive friction material) to FEP (named as negative material) due to their different electron-attracting ability, resulting in an activated TENG 1 with the same amount of positive or negative charges appeared on the FEP and the top of Cu materials, as shown in Figure 2A_1_. When the vibration film moved down driven by the wind, a separation of top Cu and FEP film was achieved. For the electrostatic induction, the electrons flowed from Cu of vibration film to the top of Cu and generated an electric current in the external circuit in TENG 1. Meanwhile, owing to the change in distance between coils and magnets, an AC signal was obtained in EMG 1 and EMG 2 based on the Faraday electromagnetic induction effect. At state 3 in Figure 2A, the bottom of Cu was contacted with FEP, and there were no output signals in TENGs and EMGs. Conversely, the FEP film moved back accompanied by the flow back of electrons in the opposite direction, and the output signals could be observed in TENGs and EMGs, as illustrated in Figure 2A_4_. As a word, AC output signals could generate in TENGs and EMGs during the contact and separation process of FEP vibration film with Cu electrodes.

The output capability of the EMG/TENG be evaluated by the open-circuit voltage (*V*_*oc*_), the short-circuit current (*I*_*sc*_), and power density. Various tribo-materials possess different triboelectric charges density; the performance of TENGs is closely related to the materials. Usually, TENGs make of at least two tribo-materials, which should readily produce triboelectric charges as well as have the opposite triboelectric polarities. According to the previous investigation, polytetrafluoroethylene (PTFE), Kapton, FEP, polyvinylidene fluoride (PVDF), and polydimethylsiloxane (PDMS) are widely utilized as negative friction material of TENG (Gao et al., 2020; Zhang et al., 2014; Zhao et al., 2020a, 2020b). Conversely, metal foil ITO, FTO, wool, wood, paper, and metal micro-nano structure are served as positive material (Chen et al., 2018; Jiang et al., 2017; Shao et al., 2017; Zhang et al., 2015). Previous studies have demonstrated that the larger surface contact area of friction material has the more excellent output property of TENGs. To maximize the output capability of the TENG, nano-structural component techniques and surface modification were implemented to enhance the roughness or the total contact area of the friction interface (Gao et al., 2019; Jiang et al., 2017; Quan and Yang, 2016a; Zhao et al., 2017, 2019).

#### The principle of PENGs

In 1880, Curie brothers P. and J. Curie first observed that external forces were applied with a particular direction to the crystals, which caused the dielectric polarization in the crystal with negative and positive charges on two opposite surfaces of these crystals. This characteristic phenomenon was known as the piezoelectric effect. The resulting electric dipole moments generated by the piezoelectric effect can be calculated by $P_{piezo}=d\cdot F$, where d refers to the piezoelectric constant and F is the external force applied on the piezoelectric materials. The PENG-based piezoelectric effect possessed prominent mechanical energy harvesting properties and could generate electrical and mechanical energy directly (Qin et al., 2008a, 2008b; Wang et al., 2016b; Wang, 2008; Wang and Wu, 2014; Xu et al., 2010; Yang and Wang, 2015; Yang et al., 2013c). Figure 2B is a detailed schematic illustration of the electrical-signal-generating mechanism of ZnO nanowires (NWs)-based PENG (Wang, 2008). As an initial state, the Si-tip-coated Pt film is located beside the pure ZnO NWs in a separation state. With Pt film coated, the Si tip was utilized as the unpliant and conductive tip, which could curve the ZnO nanowires. The morphology of surface and local external force could change the tip's height; under the external pressure, the bare ZnO NWs at the bottom bent to the right as the top tip stretches to the right. A piezoelectric signal has been generated on the whole uncovered ZnO NWs, and there were positive and negative electrical potential, respectively on the stretched and compressed surface of the ZnO NWs. Due to the reverse-biased Schottky barrier, there was no current flow between the positive potential surface and Pt-coated surface. Simultaneously, the electrons could flow through the negative potential side to Pt surface due to the forward-biased Schottky barrier when the bare ZnO NWs contact with another Pt-coated side. With the further moving, more Pt tips wiped across the uncovered ZnO NWs, generating an additional piezoelectric output for the forward-biased Schottky barrier. To obtain more electrical efficient production, the contact between tip and ZnO NWs is required for Schottky to maintain the connection with the NW, whereas the substrate is ohmic contact. In addition to ZnO, PZT, BaTiO_3_ (BTO), PVDF, and other star materials could be served for constructing PENGs with the same basic model and mechanism. Due to the inorganic piezoelectric materials with high hardness, the devices-based piezoelectric materials are easy to be damaged, thus the performance or lifetime of the PENG will be influenced. Therefore, the construction of a PENG with high stability and flexibility is an indispensable research hotspot for the development of PENG. Some meaningful referential work will also be discussed later in this review.

### The mechanism of harvesting solar energy

The annual solar radiation on earth is comparable to the energy of 13 billion tons of standard coal, about 2 × 10^4^ times global energy consumption. Abundant solar energy as environmental and sustainable energy has invariably caught the attention of researchers. The direct utilization of solar energy is divided into photothermal and photoelectric conversion. The solar thermal conversion could convert solar radiation into heat and then generate electrical output based on a thermoelectric conversion system. Photovoltaic devices are considered to be the most promising energy conversion technology in the next decade because they can convert solar energy into electricity directly based on the photovoltaic effect (Dudem et al., 2016; Li et al., 2020; Liu et al., 2018b; Wang et al., 2016a; Wei et al., 2020; Zhang et al., 2016b). After decades of creative progress, there has been a lot of mature achievement around the solar energy collection technology based on the photothermal and photoelectric effect. These various applications will be discussed in detail in subsequent sections of this review. At first, the fundamental principle of solar energy utilization technology should be summarized.

The photoelectric phenomenon was first discovered by chance, whereas Hertz experimented with Maxwell's electromagnetic theory in 1887. In 1905, Einstein gave a comprehensive explanation of the photoelectric effect by putting forward the quantum theory of light and won the Nobel Prize for Physics in 1921. The photovoltaic effect schematic illustration mechanism was as shown in Figure 2C (Zhang et al., 2017). When a photon with an energy higher than the gap width of the semiconductor incident on the semiconductor, the electrons in the semiconductor valence band could be activated to the conduction band, generating photon and electron-hole pairs in the semiconductor, simultaneously; if there is an existing asymmetric electrostatic potential in the semiconductor, the photon and electron-hole pairs will be separated under the effect of an electric field to form free electrons and holes and move toward both ends of the electric field, respectively, thus generating a photocurrent.

## The classification of harvesting mechanical energy

Widespread and different mechanical energy types in our daily life such as vibration, the wind, human motion, flowing water, waves, and rotating tire are wasted and ignored generally. TENGs based on vibratile structure, rotational structure, slidable structure, and other typical architecture were prepared to meet mechanical energy in varying conditions to induce the contact-separation of friction materials, thus provoking the triboelectrification and output the electricity. Different types of mechanical energy have different characteristics in amplitude, frequency, and location of vibration. For example, human movements' mechanical energy tends to be irregular and low frequency, whereas wind energy has a larger amplitude and disturbance. Researchers devoted themselves to constructing various TENG with different configurations to satisfy various mechanical energy collection needs. Various TENGs or PENGs are broadly divided into the following types according to harvesting other forms of mechanical energy and applied to different application scenarios: wind-based TENG (Jiang et al., 2017; Liu et al., 2018a; Wang, 2014; Wu et al., 2014; Zhao et al., 2017, 2019, 2020b), water-drops-based TENG (Dudem et al., 2016; Jeon et al., 2015; Jiang et al., 2017; Liu et al., 2018b; Roh et al., 2020; Wang, 2014; Yang et al., 2013a; Zheng et al., 2015), human-movements-based TENG (Pu et al., 2016; Qin et al., 2008a; Song et al., 2019; Wang, 2014; Wen et al., 2016; Zhang et al., 2015), wave-kinetic-energy-based TENG and PENG (Lee et al., 2010; Qin et al., 2008a; Shao et al., 2017; Yang et al., 2020). A detailed overview of these would be provided next.

### Hybridized nanogenerators with wind-based TENG

Wind energy is an environmentally friendly, widely distributed renewable energy. According to the data in Figure 1C, the increment of power generation is driven by renewable energy, of which 49% is generated by renewable energy. By 2050, about 31% of the 49% of the power generation will be generated by wind energy. Traditional wind scavenging technology is almost impossible to be used in our daily life in the urban region for many disadvantages such as large size, high cost of fabrication, geographical conditions, and relatively sizable actuating wind speed. Wind-based TENG is a favorable wind-generating technology with the superior characteristic of easy-preparation, low-cost, simple construction. It can be utilized in the conditions of environmental change. The wind-based TENG possesses a rotational structure or flutter-driven structure generally. The wind energy provokes the rotor's rotation and the contact separation of friction materials in rotational and vertical flutter structures separately to generate electrical power based on the friction electrification effect.

**Figure 3:**
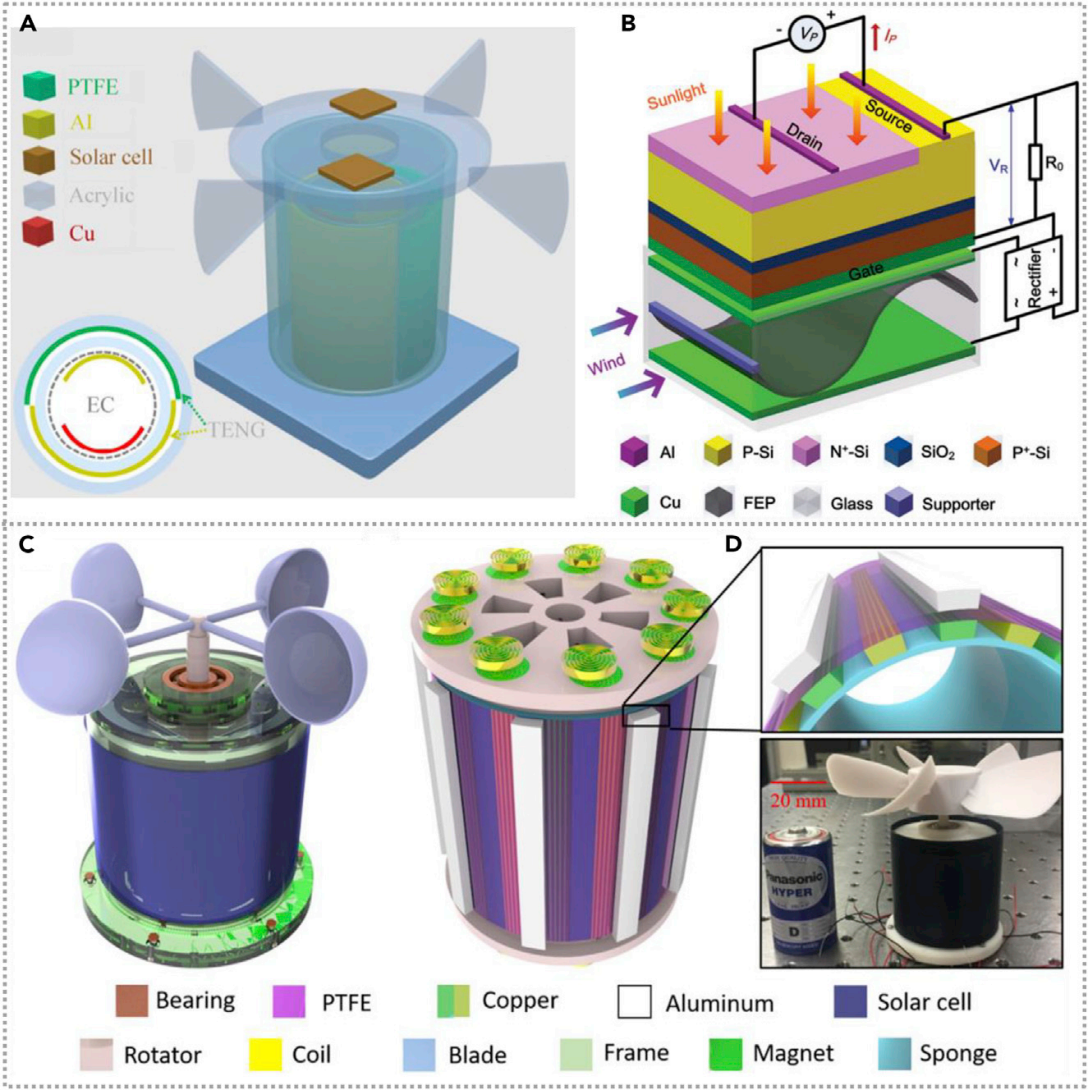
Hybridized nanogenerators with wind-based TENG (A) Schematic diagram of the fabricated hybridized energy cell. The inset shows the cross-sectional schematic diagram of the device. Reproduced with permission, from ref (Wu et al., 2014), Copyright 2014, Springer. (B) Schematic illustration of coupled energy harvester, the TENG, enhancing the solar energy conversion. Reproduced with permission, from ref (Zhang et al., 2016a), Copyright 2016, Wiley-VCH. (C) Schematic illustration of the functional components of device, which mainly consists of one TENG, two EMG groups, and a commercial W-SC. Enlarged view of the detailed structure of TENG unit. Reproduced with permission, from ref (Qian and Jing, 2018), Copyright 2018, Elsevier. (D) An optical image of the fabricated device and commercial battery is used to compare with the device dimension. Reproduced with permission, from ref (Qian and Jing, 2018), Copyright 2018, Elsevier.

Qian et al. constructed a self-powered natural disaster monitoring system based on a hybridized harvester, which consists of eighteen EMGs, one TENG, and one flexible solar cell (Qian and Jing, 2018). The enlarged schematic illustration and structure photograph of that multifunctional generator is as shown in Figures 3A and 3B. The energy cell could deliver great electricity in the wind field, the output property of TENG and EMG enhanced with the increasing of rotation speed and the performance of EMG and TENG without apparent attenuation after thousands of cycles. The generating electricity of fabricated hybridized energy cells could be stored in a commercial capacitor, using rectifier and transformer to power various sensors in large-scale arrangements to monitor natural disasters. A self-powered monitoring system was constructed successfully. Coincidentally, a hybrid energy harvester was designed to simultaneously generate wind and solar energy (Wu et al., 2014). The schematic diagram of the fabricated hybridized energy cell is shown in Figure 3C, and the inset shows the cross-sectional schematic diagram of the device. The PTFE and Al film could contact and separate with each other periodically driven by the wind based on a rotatable structure. The TENG could deliver an output of 90 V/0.5 mA/m^2^, and the total electricity of the hybrid nanogenerator could play a role as a power unit for personal electronic devices. Wind energy can complement solar energy harvesting and improve solar energy conversion (Zhang et al., 2016a). This work is based on the field-effect phototransistor and TENG with a creative significance, and the schematic diagram of the principle and structure of the coupled energy collector is shown in Figure 3D. The power of the solar energy harvester could be substantially enlarged and increased monotonously under different wind speeds, and the power of the solar cell is only 231 nW, whereas the hybridized cell with 15.4 μW is under 12 m/s. This work demonstrated a promising multiple-effects-coupled energy generator to deliver a larger output signal by scavenging different energy types.

### Hybridized nanogenerators with water-drops-based TENG

In addition to wind energy, the raindrops or other liquid drops will also cause friction on the contact surface when they flow down, resulting in friction electrification phenomenon. TENG could utilize the power of water drops or raindrops with a simple but ingenious structure, which is a complement to solar energy or wind energy. Moreover, waterdrops could help clear the dust on the surface of TENG to maintain a more stable performance and prolong the lifetime and robustness of TENG.

**Figure 4:**
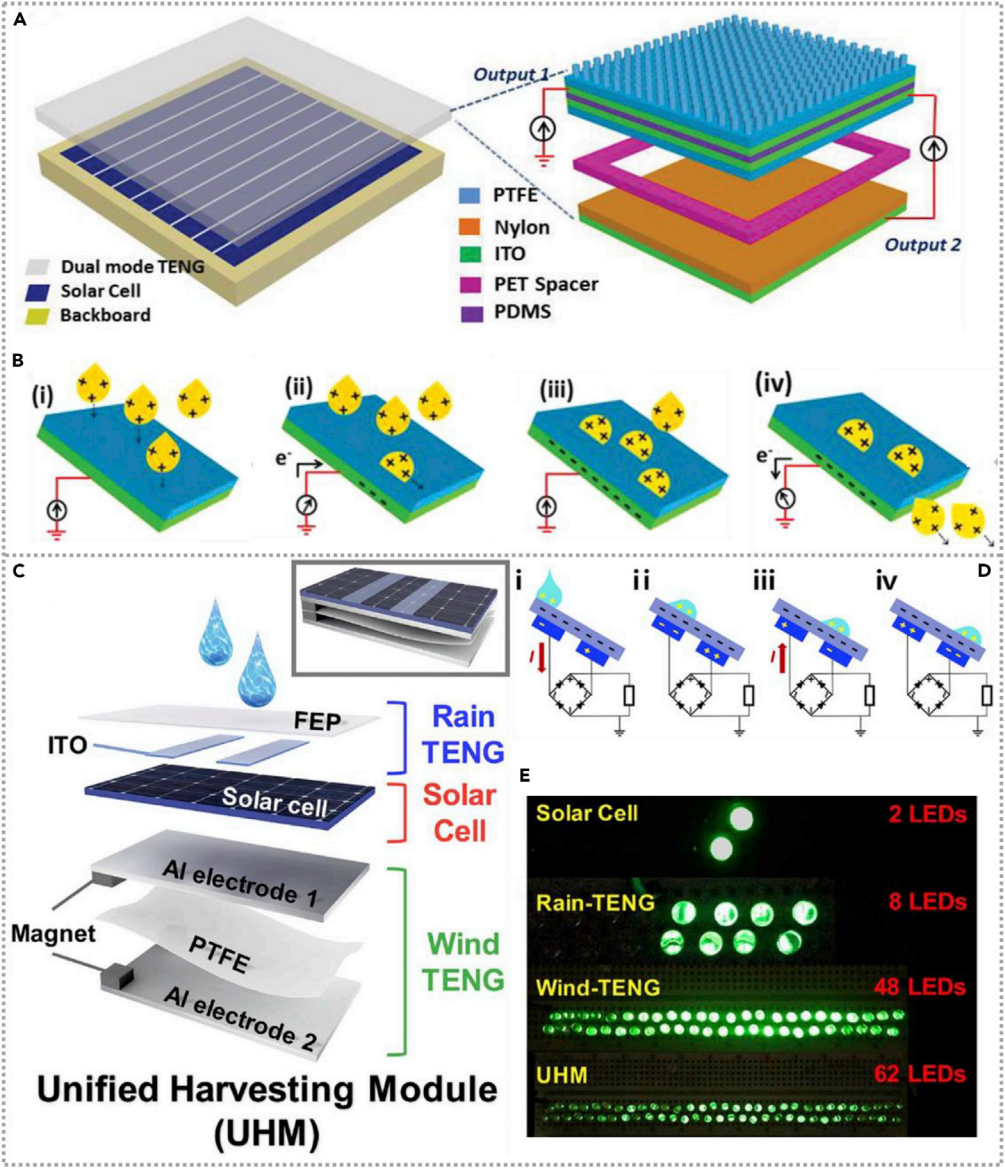
Hybridized nanogenerators with waterdrops-based TENG (A and B) Schematics of the unified harvesting module and an enlarged view of the dual-mode TENG in detail and the rain-TENG working mechanism. Reproduced with permission, from ref (Zheng et al., 2015), Copyright 2015, Wiley-VCH. (C) The schematics diagram of hybridized energy cell consists of rain-based TENG, solar cell, and wind-based TENG from top to bottom. Reproduced with permission, from ref (Roh et al., 2020), Copyright 2020, Elsevier. (D) The working mechanism of the water TENG. Reproduced with permission, from ref (Roh et al., 2020), Copyright 2020, Elsevier. (E) Green LEDs could be light by a single solar cell, rain TENG, wind TENG unit, or the whole device. Reproduced with permission, from ref (Roh et al., 2020), Copyright 2020, Elsevier.

In 2015, Zheng et al. developed a cost-effective hybridized energy panel (Zheng et al., 2015) in an integrated energy unit combined with dual-mode water droplets based on TENG with wind energy and the solar cell to expand the scavenging scope of wind, solar, and rain energy. The construction of that hybridized cell is shown in Figure 4A and exhibits the enlarged view of the dual-mode TENG in detail with the working mechanism of water-based TENG in Figure 4B. The triboelectric charges generated in waterdrops due to the contact electrification of drops with the surface material, the polarity, and density of charges on drops depend on the friction material and weather condition. Under different conditions, water- and wind-based TENG could generate 86 mW/m^2^ and 8 mW/m^2^ separately. In addition to harvesting energy, a lightweight and ultrathin multifunctional sensor could be established based on the hybridized energy panel. In 2020, researchers fabricated an energy cell to scavenge raindrops, solar, and wind energy and deliver a superior electrical output, whether rainy, sunny, or windy (Roh et al., 2020). Figure 4C is the schematics diagram of that hybridized energy cell, consisting of rain-based TENG, solar cell, and wind-based TENG from top to bottom. The operating mechanism of rain-based TENG exhibits in Figure 4D**.** The output of rain-based TENG is affected by the angle of the TENG unit and the PH of the liquid solutions, indicating rain TENG could be utilized as a sensor for acid rain. The hybridized energy cell could be actuated under different conditions. Whether a single solar cell, a rain TENG, a wind TENG unit, or the whole device could be used as a power unit to light green LEDs, and their photos are exhibited in Figure 4E. The energy cell could play an essential role as a self-powered multifunctional device to collect energy in our surroundings and monitor the weather in real-time simultaneously.

### Hybridized nanogenerators with human-movements-based TENG

As a kind of mechanical energy widely existing in our daily life, the energy concomitant with human movements is being ignored and wasted generally. The wide range of humans' activities could generate mechanical energy such as walking, running, waving arms, shaking hands, and so on. Constructing human movements based on TENG is a valuable energy-generating technology to deal with the global energy crisis. It could play an important role in artificial intelligence and wearable healthcare-related equipment. The human-movements-based TENG is flexible and lightweight in general, and friction materials can be contacted by human motion and separated by human activities (Chen et al., 2016; Ma et al., 2019; Pu et al., 2016; Song et al., 2019).

**Figure 5:**
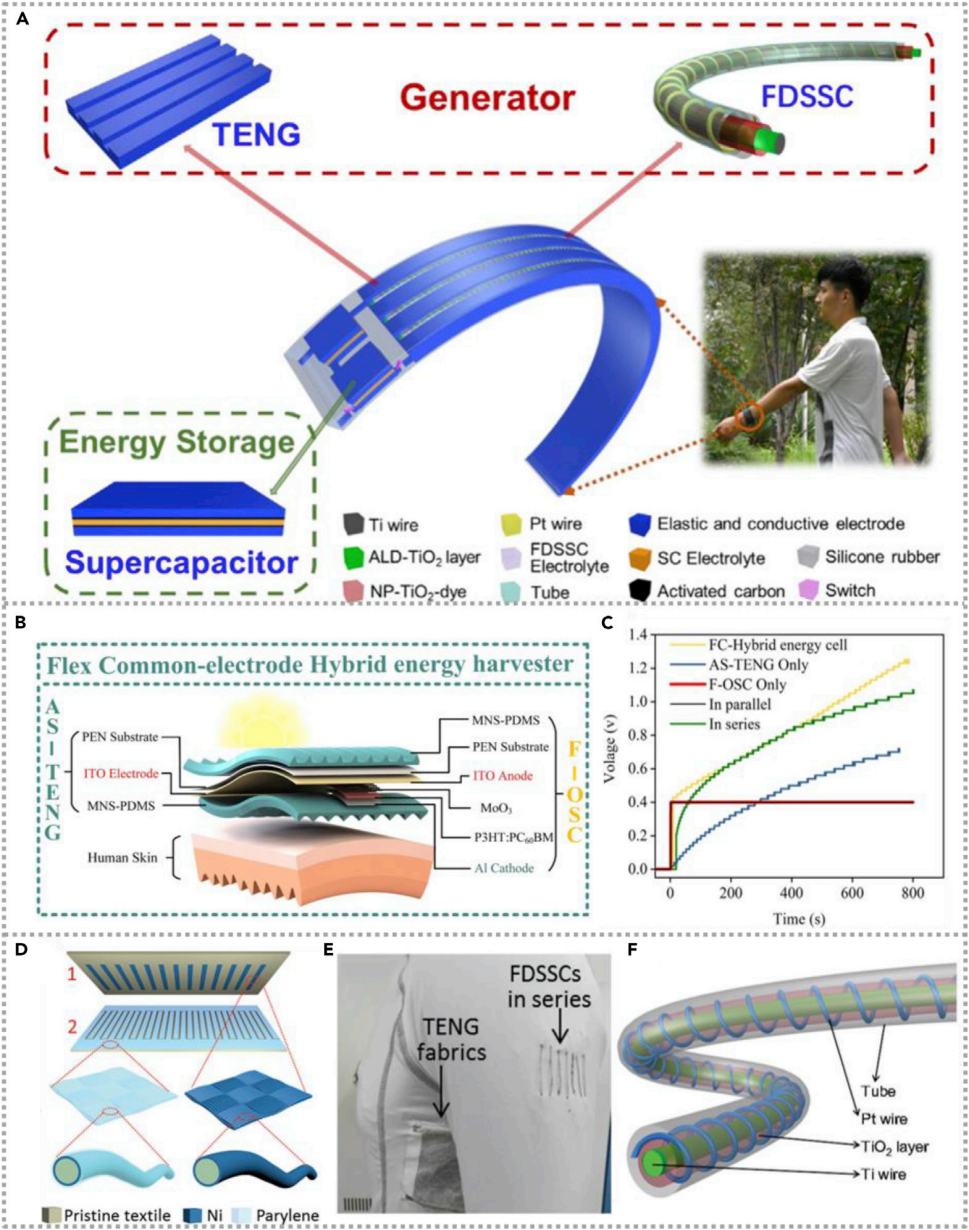
Hybridized nanogenerators with human-movements-based TENG (A) Schematic of the self-charging power bracelet, the fiber-shaped dye-sensitized solar cell, and TENG exhibit in detail. Reproduced with permission, from ref (Song et al., 2019), Copyright 2019, Elsevier Ltd. (B and C) Schematic illustration of a flexible hybrid system and the charging curves of the capacitor with different energy units. Reproduced with permission, from ref (Ren et al., 2020), Copyright 2019, Elsevier Ltd. (D–F) The TENG fabrics configuration, a power-textile photo with a pair of TENG fabrics and seven DSSCs in series, and construction of fabric-based DSSC. Reproduced with permission, from ref (Pu et al., 2016), Copyright 2016, Wiley-VCH.

To meet human movements' application characteristics, Song et al. developed a highly elastic wearable energy device to scavenge solar energy and human actions and integrated with a supercapacitor to store electricity (Song et al., 2019). The stretchable and comfortable hybridized energy system could be utilized as a self-charging bracelet to scavenge the wrist movement and solar energy in our daily lives. Figure 5A exhibits the construction of a self-powered bracelet consisting of TENG, a flexible dye-sensitized solar cell, and a storage supercapacitor. The bracelet's performance without obvious decrease after 10,000 cycles of bending tests and the whole energy harvesting device could employ as a stable power unit for an electronic watch. Similarly, a novel flexible self-cleaning hybrid energy harvesting system was constructed, including a single-electrode triboelectric nanogenerator and a flexible organic solar cell (OSC) and could be attached to humans' cloth to scavenge the energy from the movement of the arm (Ren et al., 2020). The schematic illustration of the fabricated hybrid energy cell is shown in Figure 5B. The solar energy and mechanical energy from the top and bottom parts do not interfere with each other, thus the hybrid energy cell could simultaneously harvest both solar and mechanical energy through two separate energy units. Figure 5C exhibits the charging curves of the 10 μF capacitor charged by a single TENG, OSCs, and hybrid energy cell, respectively. The hybrid energy cell could deliver greater output than the independent energy unit. The flexible hybrid energy cell possesses dust-proof, self-cleaning, and self-encapsulation characteristics, resulting in a stable performance even under extreme water erosion conditions.

In addition to flexible planar hybridized energy cell, textile-shaped energy harvester has superior advantages, some of them being easy integration, large scale, and excellently breathable. Pu et al. prepared wearable energy textiles consisting of fabric TENG and fiber-based DSSC to scavenge human movements and solar energy simultaneously and separately (Pu et al., 2016). The configuration and photograph of the textile-based TENG and DSSC are exhibited in Figures 5D and 5F, showing the fabric-shaped devices could be integrated in people's clothing easily. The hybridized energy cell could obtain the output of 3.2 W/m^2^ and 10.6 mA/cm^2^ based on TENG and DSSC separately, indicating its superior energy collecting property and promising to apply in self-charging electric devices area.

### Hybridized nanogenerators with wave-kinetic-energy-based TENG

Wave energy, especially ocean/water wave energy(also called blue energy), is abundant and extensive, which is quite challenging to collect through EMG for low-frequency and irregular amplitude. It is necessary to design an efficient, environmentally friendly and inexpensive wave energy harvesting technology (Chen et al., 2020; Shao et al., 2017; Yang et al., 2019; Yang et al., 2020). The structural design is particularly important to make great use of wave energy in our environment.

**Figure 6:**
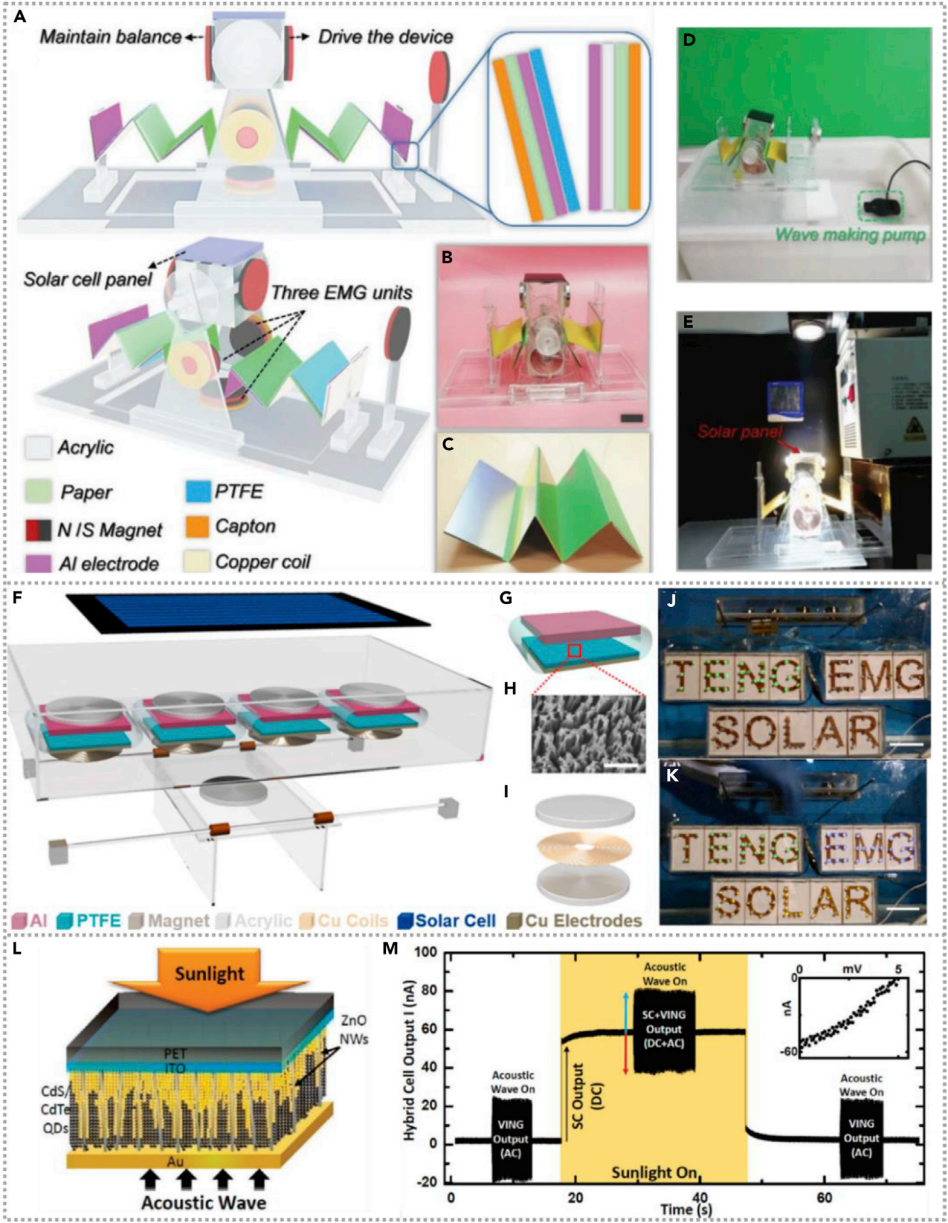
Hybridized nanogenerators with wave-kinetic-energy-based TENG (A–C) Structural diagram and photograph of the hybridized generator as well as the photo of one zigzag multilayered TENG. Reproduced with permission, from ref (Yang et al., 2019), Copyright 2019, Wiley-VCH. (D and E) The hybridized energy cell is used as a practical power source in the environment. Reproduced with permission, from ref (Yang et al., 2019), Copyright 2019, Wiley-VCH. (F–K) Schematic illustration of the hybridized power unit and the power cell could be utilized as a power source at different conditions. Reproduced with permission, from ref (Shao et al., 2017), Copyright 2017, Elsevier Ltd. (L and M) Schematic of a nanowire-quantum dot hybridized cell and the current signal of hybridized energy unit. Reproduced with permission, from ref (Lee et al., 2010), Copyright 2010, American Chemical Society.

The seawater immersion and corrosion and the package are a challenge for the development of blue-energy harvesters. The solar energy on the surface of the ocean should be complementary to wave energy. A pendulum-like paper-based hybridized nanogenerator was designed exquisitely to scavenge low-frequency water wave and solar power (Yang et al., 2019), the pairs of magnets driven by water wave could operate the TENG efficiently. The diagram of the structure and optical photo of the hybridized generator are shown in Figures 6A and 6B, and the picture of one zigzag-shaped multilayered TENG is shown in Figure 6C. The hybridized energy cell could be served as a valuable power source driven by water wave or solar illumination and that energy unit as exhibited as Figures 6D and 6E. The TENGs of devices could provide a maximum power of 22.5 mW, and the solar panel could continuously supply power for the thermohygrometer; moreover, the prepared hybridized cell could be driven by hand movement.

A promising hybridized nanogenerator constructed by H. Shao et al. could efficiently collect blue energy and solar energy. Several contact-separation mode TENGs and freestanding sliding mode EMGs were employed in hybridized energy cells to efficiently scavenge water wave energy and a water-proof solar cell to harvest solar energy simultaneously (Shao et al., 2017). The schematic illustration of the typical hybridized power unit is shown in Figure 6F and the single TENG and EMG shown as Figures 6G–6I, and Figures 6J and 6K show the hybridized power cell can be used as a power supply to light LEDs at low-frequency motion without solar illumination and high-frequency movement with light, respectively. The prepared hybridized energy cell could play the role of sustainable power unit even under low-frequency waves.

Besides water wave energy, sound wave energy could also be scavenged by nanogenerators; researchers developed a nanowire-quantum dots hybridized cell to utilize sound wave and solar energy (Lee et al., 2010). The schematic diagram of the energy harvester is shown in Figure 6L, the solar illumination and sound vibration are spreaded from the top surface PET/ITO material and the bottom of Au electrode plate, respectively. The output performance of the nanogenerator could be influenced by the input acoustic energy, and the energy device could also scavenge solar energy combining with CdS/CdTe quantum dots in it. Figure 6M demonstrated the current output signal of the hybridized energy unit when the acoustic wave was applied to the energy cell during and after solar illumination, indicating hybridized energy unit could generate solar and acoustic energy individually or simultaneously in our daily life.

### Hybridized nanogenerators with piezoelectric nanogenerator

**Figure 7:**
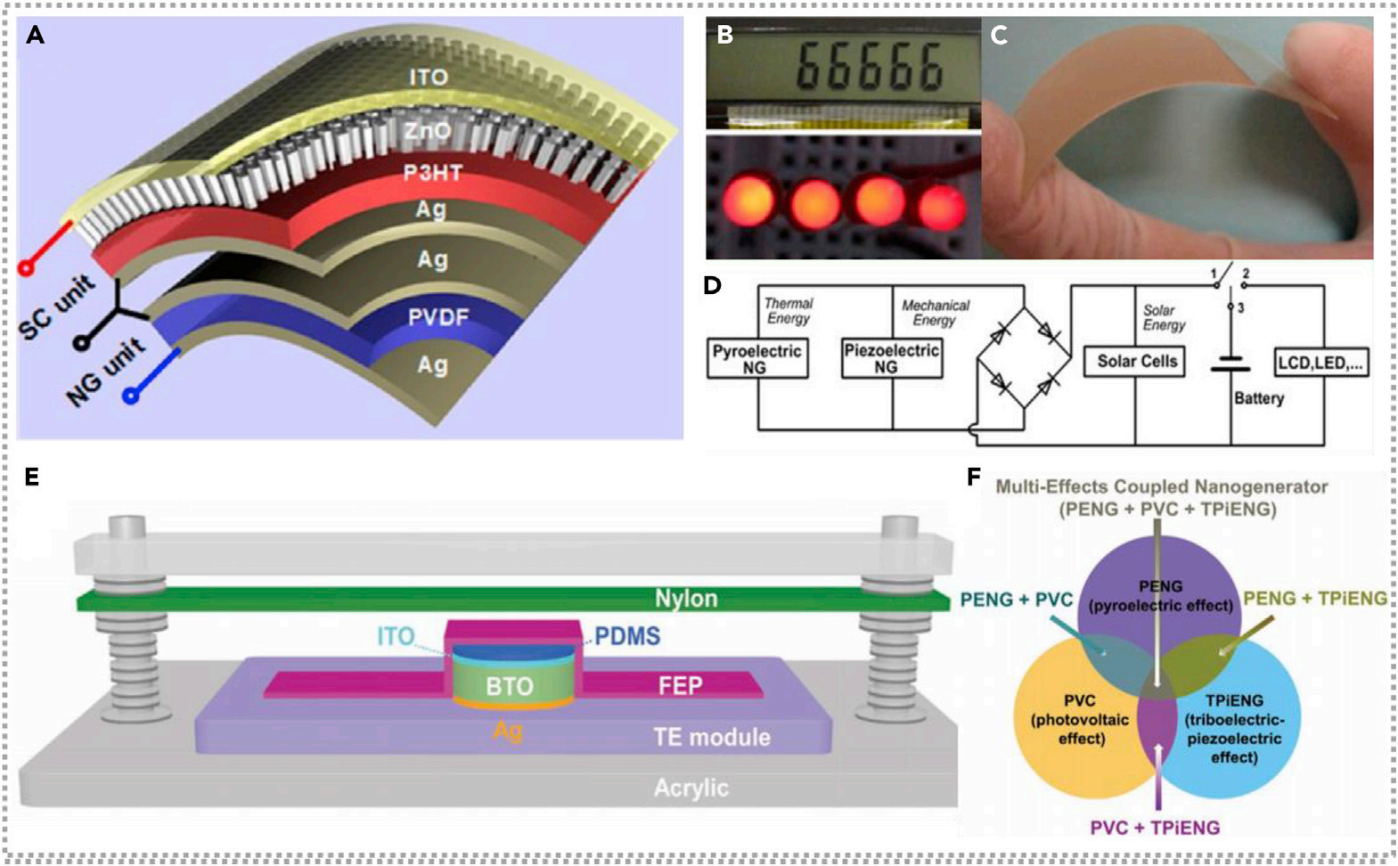
Hybridized nanogenerators with piezoelectric nanogenerators (A) Schematic diagram of the fabricated hybridized energy cell. Reproduced with permission, from ref (Yang et al., 2013c), Copyright 2013, American Chemical Society. (B–D) Optical picture of LEDs driven by the charged Li-ion battery with hybridized energy cell, a photograph of the bent ZnO nanowire on a flexible ITO/PET substrate, and equivalent circuit diagram of hybridized energy unit. Reproduced with permission, from ref (Yang et al., 2013c), Copyright 2013, American Chemical Society. (E) The structural illustration of a hybridized nanogenerator combining piezo-tribo-pyro-photoelectric effects. Reproduced with permission, from ref (Ji et al., 2018), Copyright 2017, Wiley-VCH. (F) The design-operating principle of coupled nanogenerator combining piezo-tribo-pyro-photoelectric effects. Reproduced with permission, from ref (Ji et al., 2018), Copyright 2017, Wiley-VCH.

Yang et al. fabricated a fully flexible hybridized energy unit combined with a pyroelectric NG (Yang et al., 2013c), a piezoelectric NG, and a solar cell, scavenging thermal, mechanical, and solar energy individually or simultaneously. Figures 7A and 7C demonstrate the schematic illustration and super flexibility of the prepared hybridized energy device, respectively. As shown in Figure 7B, the mixed energy cell could be utilized as the power source for lighting the LCD through hand-touch induction. In addition, the pictures of the four red LEDs are lighted by a Li-ion battery charged via a hybridized nanogenerator, and the equivalent circuit diagram of that energy cell is demonstrated in Figure 7D. A multi-effect-coupling nanogenerator based on ferroelectric barium titanate has fabricated in 2018 (Ji et al., 2018). It could simultaneously scavenge thermal, solar, and mechanical energy, the structural illustration and design operating principle of fabricated hybrid nanogenerator, as shown in Figures 7E and 7F, by integrating thermoelectric, photovoltaic cells and a triboelectric-piezoelectric nanogenerator in a whole structure with only two electrodes. Compared with the traditional stacked hybrid nanogenerator, the multi-effect coupling nanogenerator based on one configuration is smaller in size, simpler in construction, and lower in cost. At the heating rate of 0.98 K/s, 405 nm LED lighting, and airflow speed of 15 m/s, the hybrid energy could deliver a peak output of 1.5 μA/7 V with a 6 V platform voltage, which could charge a 0.33 μf capacitor to 1.1 V easily in 10 s. It has a broad practical application prospect and represents a new trend of integrated multi-energy recovery.

## The classification of harvesting solar energy

Solar photovoltaic conversion technology has developed several critical historical stages since its development. In 1883, the first semiconductor selenium photovoltaic cells with photovoltaic efficiency of 1% were produced. By 1954, Bell Labs created the first monocrystalline silicon solar cell with a conversion efficiency of 5%. Researchers have been exploring gradually until now. Various types of solar energy collectors have been constructed, including commercial solar cells, OSCs, dye-sensitive solar cells, flexible solar cells, heterojunction solar cells, and solar-thermal pyroelectric generators. Different types of solar energy conversion technologies have other characteristics and application conditions; all these technologies and mechanical energy harvesting technologies can be integrated into hybridized nanogenerators through structural design to collect solar and mechanical energy simultaneously.

### Hybridized nanogenerators with conventional solar cell

As an essential photovoltaic technology with high photoelectric conversion efficiency, crystalline silicon solar cells have a market share of more than 90%, which will remain the mainstream type of solar energy collector for a long time in the future.

**Figure 8:**
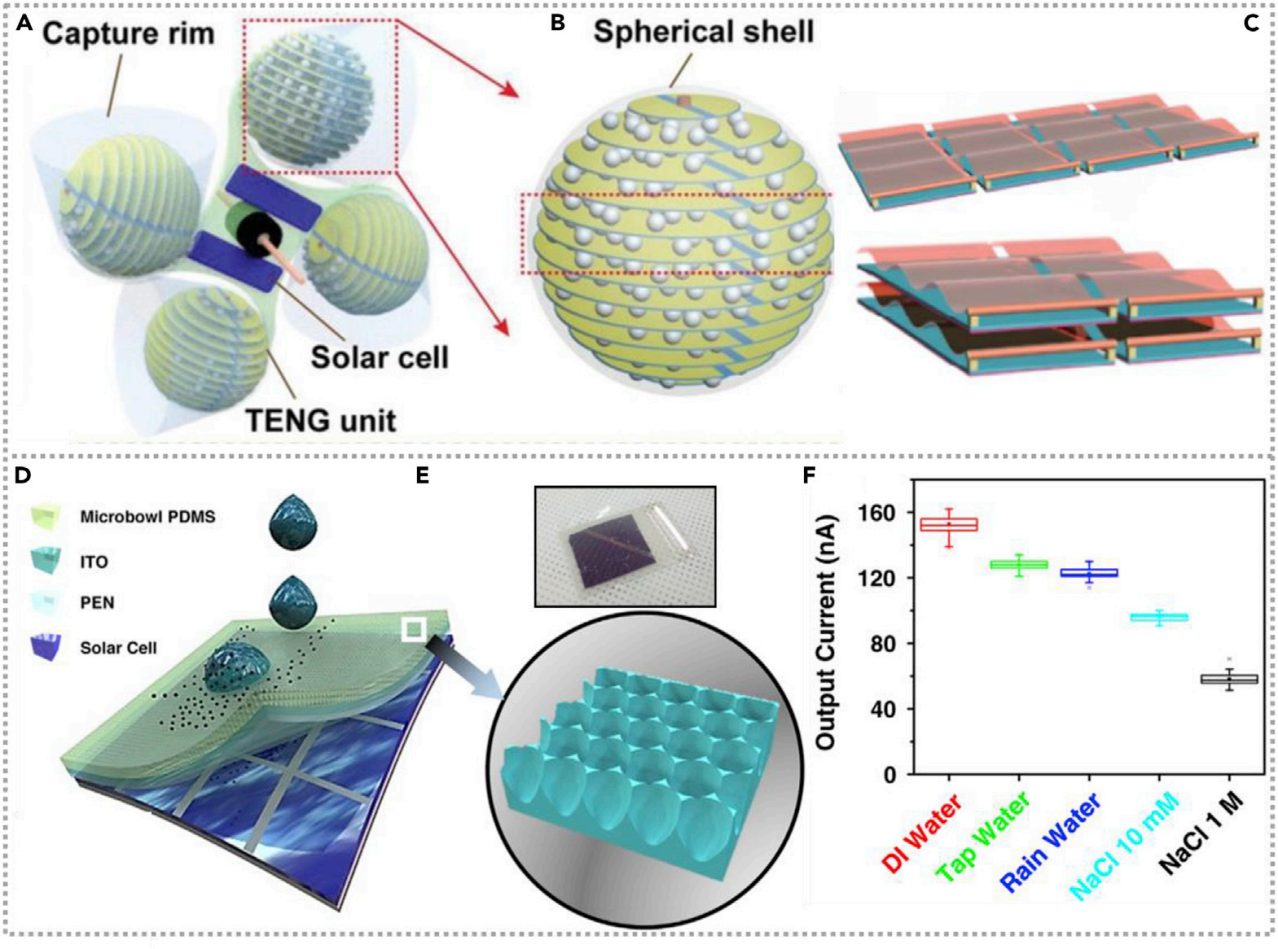
Hybridized nanogenerators with a conventional solar cell (A and B) The hybrid all-in-one power source's unique structure for harvesting multiple environmental energy types and the TENG unit's enlarged construction with multilayer architecture. Reproduced with permission, from ref (Xu et al., 2020), Copyright 2020, Wiley-VCH. (C) Schematic diagrams of a flag-like hybridized nanogenerator and the 4 × 1 TENG arrays and 2 × 2 TENG arrays. Reproduced with permission, from ref (Wang et al., 2017), Copyright 2017, Elsevier. (D and E) Schematic of hybridized cell and a magnified view of the PDMS with the macro bowl array and a digital photograph of that hybridized cell. Reproduced with permission, from ref (Jeon et al., 2015), Copyright 2015, Elsevier. (F) Statistics of the output currents generated by different kinds of the liquid droplet. Reproduced with permission, from ref (Jeon et al., 2015), Copyright 2015, Elsevier.

An efficient hybridized nanogenerator comprises commercial solar cells and TENG (Wang et al., 2016a), which could be widespread installed on the roof of buildings and scavenge solar and wind energy simultaneously. Under wind speed of 15 m/s and full-sun illumination, the TENG and SC could provide a 375 V/260 μA/26 mW and 7 V/9 mA/8 mW output, respectively. The energy captured by the fabricated hybridized nanogenerator could drive the sensor quickly and directly and charge the commercial Li-ion battery, which gives a meaningful prospect for the practical applications of collection renewable energy. A hybrid all-in-one energy cell consisting of spherical triboelectric nanogenerators and commercial solar cells was demonstrated to scavenge most typical environmental energies from wind, raindrops, and sunlight (Xu et al., 2020). Figures 8A and 8B exhibit the rational structure of fabricated all-in-one hybrid energy cell and the TENG unit with a multilayer structure. Under the wind, the hybrid energy cell could deliver almost continuous DC with a high average power of 5.63 mW and could light 1,160 LEDs. By complementing the solar cell, the whole energy unit could be employed as a reliable power source even at unstable environmental energy conditions, and self-powered soil moisture control, forest fire prevention, and pipeline monitoring could be demonstrated. Jie Wang prepared a flag-shaped hybridized nanogenerator with a simple design structure to independently scavenge wind and solar energy (Wang et al., 2017). Fabricated NG could be easily integrated into the arrays; Figure 8C exhibits the schematic diagrams of single TENG, the 41 TENG array, and 2 × 2 TENG array. Subsequently, a multifunctional self-powered wind direction and wind speed sensor based on hybridized NG was produced, which gives a great potential of nanogenerator in the age of artificial intelligence.

To overcome the influence of continuous dust pollution on the commercial solar cell's performance and the shortage of solar energy in rainy days, a self-cleaning complementary hybridized nanogenerator was constructed to collect raindrops and solar power (Jeon et al., 2015). The schematic and digital photograph of the hybridized cell was shown in Figure 8D and the insert of Figure 8E. The water-based TENG has a superhydrophobic PDMS surface, such as the micro bowl array surface as shown in Figure 8E, which has high uniformity and a large surface contact area. It could generate an output of 7 V/128 nA driven by droplets above 15cm of TENG. Both the solar cell and TENG could generate electrical output respectively at one time; interestingly, the production of droplet-based TENG is affected by the type of liquid (Figure 8F), whereas the LED could light by water-based TENG.

### Hybridized nanogenerators with dye-sensitized solar cell

The dye-sensitized solar cells are primarily composed of a semiconductor electrode adsorbed dye-sensitized agent, electrolyte, and a Pt counter electrode. At present, the research mainly focuses on titanium dioxide thin-film material, electrolyte development, dye molecule design, and other aspects. In the meantime, how to improve the photoelectric conversion efficiency of DSSC by improving the utilization ratio of light has been a research hotspot in this field. After nearly two decades of development and optimization, the efficiency of DSSC has exceeded 13%. The DSSC is expected to develop into a potent competitor for traditional silicon-based solar cells to its superiorities of high efficiency, low cost, lightweight, and simple preparation.

**Figure 9:**
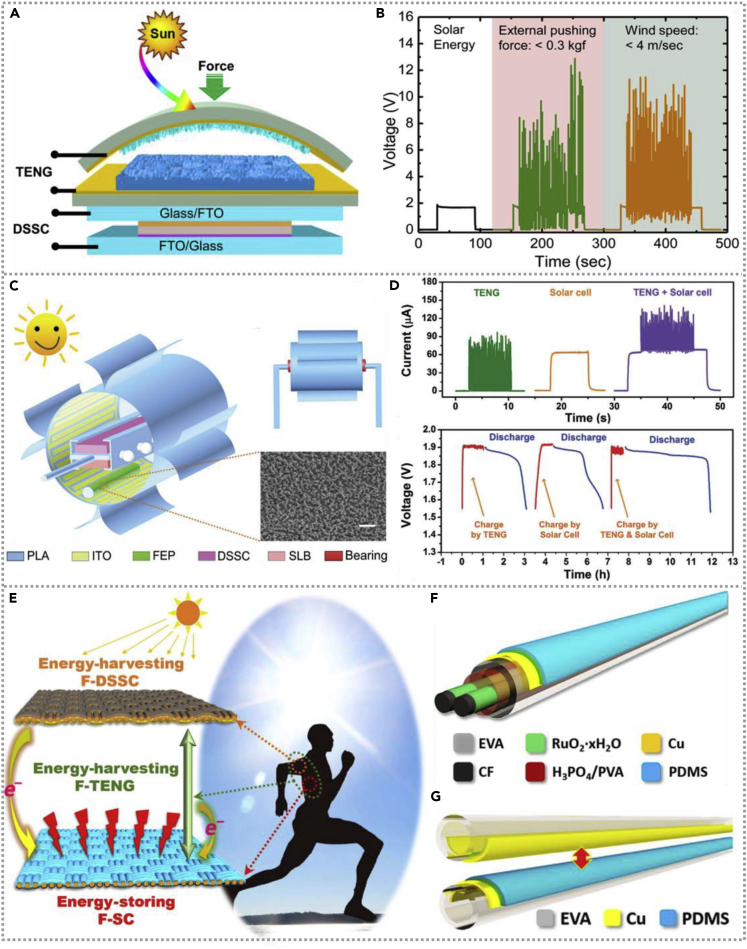
Hybridized nanogenerators with dye-sensitized solar cell (DDSC) (A and B) A hierarchical arch-shaped hybridized nanogenerator scavenging wind, solar, and mechanical energy simultaneously and the voltage under different conditions. Reproduced with permission, from ref (Dudem et al., 2016), Copyright 2016, American Chemical Society. (C and D) The schematic illustration and the output signals of a self-powered lantern hybridized nanogenerator consist of a TENG, DSSCs, and a soft lithium battery. Reproduced with permission, from ref (Cao et al., 2018), Copyright 2018, Wiley-VCH. (E–G) Schematic of the self-charging power textile system and configuration of fiber-shaped supercapacitor and single-fiber TENG. Reproduced with permission, from ref (Wen et al., 2016), Copyright 2016, Science Advances.

A hierarchical arch-shaped hybrid nanogenerator (Dudem et al., 2016) with simultaneous removal of wind energy, solar energy, and mechanical energy was prepared and illustrated in Figure 9A. The nano/micro-architectured PDMS film component helps increase the effective contact area and the TENG's light absorption. The solar cells enhance the nanogenerator's overall capability. Figure 9B illustrates the hybrid cell consisted of four DSSCs, and the TENG in series could scavenge the solar, mechanical, and wind energy simultaneously or individually. The DSSC shows super efficiency for the available spectrum range from 400 nm to 800 nm. Under 0.5 Hz external force, the TENG could generate 18.2 V/1.4 μA output. Various electronic power storage components could be subtly integrated with nanogenerators to manufacture an efficient and complete energy harvesting system. A self-powered lantern-shaped hybrid nanogenerator consists of a TENG, DSSCs, a flexible lithium battery, and LEDs; the schematic illustration of the main section of the self-powered device is shown in Figure 9C (Cao et al., 2018). Figure 9D exhibits the Isc of the TENG, DSSCs, and the hybrid nanogenerator (TENG-DSSCs are connected in a parallel state). Compared with TENG or solar cells, the hybrid nanogenerator exhibits superior efficiency of energy collecting with 150 μA electrical signal under 200 rpm rotation and 2 mW illumination. It can be seen that hybridized nanogenerator has faster-charging speed and longer discharging time, showing that they have the highest energy capacity through the charge and discharge voltage curves of TENG, DSSC, and the lithium battery with hybrid NG.

Wearable devices have more and more extensive application prospects in the era of artificial intelligence. If wearable devices could overcome the limitation of external power supply, that will promote artificial intelligence development. Wen et al.developed a flexible self-charging energy system harvesting solar and human motion energy by integrating fiber-shaped DSSCs, TENG, and supercapacitors together (Wen et al., 2016). The schematic of this power textile is shown in Figure 9E; each DSSC and supercapacitor (SC) unit is interlaced, meanwhile, the bottom surface of DSSC and the top layer of SC forming a TENG cell. Figures 9F and 9G are the detailed structure schematics of an individual fiber-shaped DSSC and fiber-shaped TENG, respectively. The hybrid power system has superior performance and the power conversion efficiency of DSSC as high as 5.64%, whereas the TENG delivers 0.91 μA current by human jogging. The fabricated textiles-based hybrid energy system could simultaneously harvest solar and human motion energy, convert them into electrical energy, and store them in the form of chemical energy, which is of great significance for developing self-powered flexible textiles devices.

### Hybridized nanogenerators with organic solar cell and a-Si thin-film solar cell

OSCs are produced from organic matter with photosensitive properties as semiconductor materials, which directly convert solar energy to electric energy based on the photovoltaic effect. The OSC and a-Si thin-film solar cell have been attracting significant attention for their characteristics of flexibility, low preparation cost, lightweight, and high light-absorption coefficient. The flexible OSC and a-Si thin-film solar cell with a simple structure could easily integrate with TENG to construct a hybrid NG, capable of efficiently harvesting mechanical and solar energy round-the-clock.

**Figure 10:**
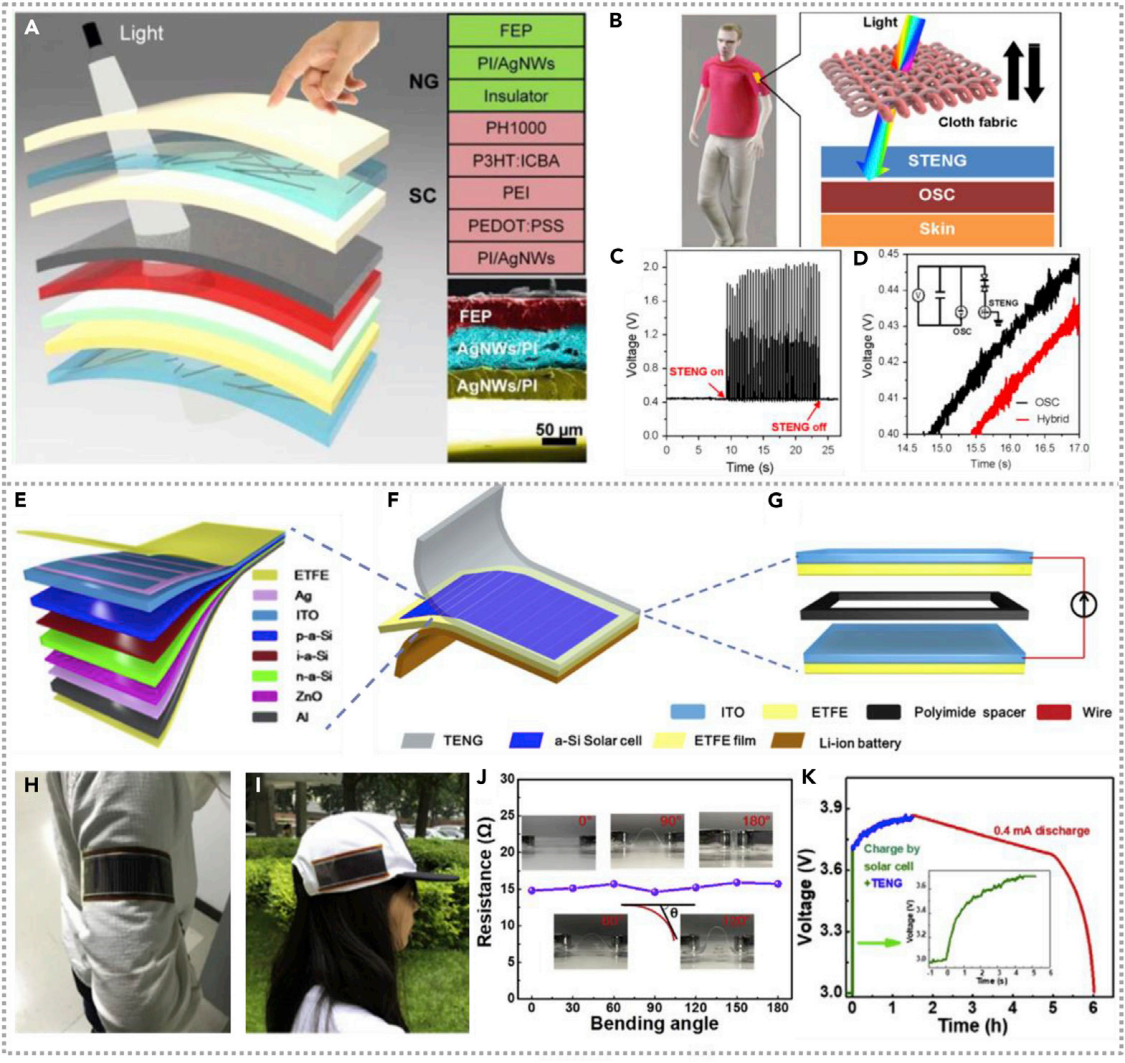
Hybridized nanogenerators with organic solar cell and a-Si thin-film solar cell (A and B) The view layout of the hybridized cell and the hybridized NG under the cloth be utilized as a wearable power source and its mechanism. Reproduced with permission, from ref (Fang et al., 2015), Copyright 2015, Elsevier Ltd. (C and D) The output of hybridized cell with and without TENG and the time spent to charge a commercial capacitor with the inset exhibits the equivalent circuit. Reproduced with permission, from ref (Fang et al., 2015), Copyright 2015, Elsevier Ltd. (E–I) The architecture of the hybridized flexible energy harvesting system and fabricated energy unit could be attached to the textiles of the arm and cap to harvest solar and mechanical energy. Reproduced with permission, from ref (Ma et al., 2019), Copyright 2019, Elsevier Ltd. (J and K) The bending test of flexible device and the charging voltage curve of Li-ion battery by energy unit. Reproduced with permission, from ref (Ma et al., 2019), Copyright 2019, Elsevier Ltd.

A flexible thin-film hybridized NG (Fang et al., 2015) that consisted of single-electrode TENG and OSC is fabricated by Yuanxing Fang and co-workers. Figure 10A exhibits the decomposition structure and view layout of the hybridized cell. The TENG is composed of FEP, PI/AgNWs, and PI film, which has excellent transparency under the light spectrum with a bit of a negative effect on the photoelectric conversion efficiency of OSC. The hybridized NG under the cloth fabric could be utilized as a wearable power source, and the schematic illustration of the mechanism is shown in Figure 10B. Figure 10C illustrates the output voltage of hybrid cell with and without TENG; the single OSC generates 0.45 V output, whereas the voltage connected to TENG is as high as 1.9 V. Similarly, the time spent on charging a commercial capacitor driven by the hybridized cell with or without TENG is shown in Figure 10D, and the inset exhibits the equivalent circuit, which demonstrates the hybrid energy cell is more effective than any single generator in energy harvesting system and could be served as a sustainable power supply for electric devices. In 2019, researchers integrated a self-powered energy system with a thickness of only 1 mm, composed of a film-shaped TENG, a-Si thin-film solar cell, and Li-ion battery sequentially from top to bottom (Ma et al., 2019). The architecture of the hybridized flexible energy harvesting system, a-Si solar cell, and the dual-mode TENG is illustrated in detail in Figures 10E–10G. From Figure 10H, there is no influence on the ETFE-ITO film's resistance at the varying bending angle (0°–180°). With superior flexibility and wearability, the fabricated energy harvesting system could be attached to the textiles of arms and caps to harvest solar and mechanical energy (Figures 10I–10J). To maximize the property of harvesting power, the energy device is connected with a Li-ion battery and the charging voltage curve of the cell by the a-Si solar cell and TENG (shown in Figure 10K); the a-Si solar cell could charge the Li-ion battery from 3 V to 3.6 V and then the battery was continued charging to 3.86 V by a TENG. Compared with a bare solar cell, the hybrid energy harvesting system with compensative significance.

### Hybridized nanogenerators with heterojunction solar cell

The heterojunction solar cell is also a promising solar energy harvesting technology, which has ample space to improve photoelectric conversion efficiency, reduce the production cost, and decrease the photoinduced attenuation. Researchers have been devoted to fabricating an integrated hybrid energy collecting system to compensate for solar cells' application conditions and power property.

**Figure 11:**
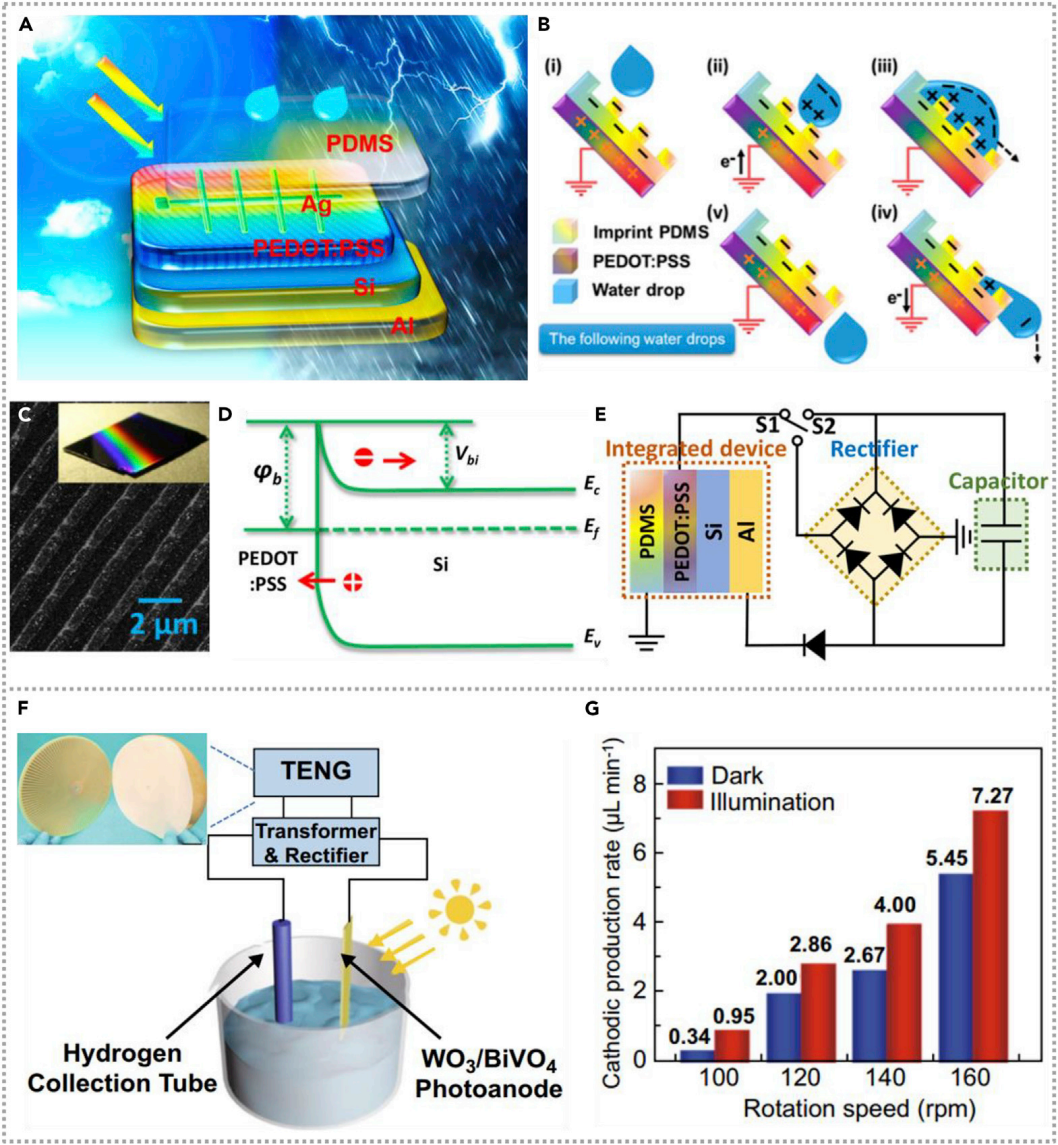
Hybridized nanogenerators with heterojunction solar cell (A and B) The demonstration of the integrated hybridized energy harvester and the working mechanism of raindrops-based TENG. Reproduced with permission, from ref (Liu et al., 2018b), Copyright 2018, American Chemical Society. (C–E) The band energy diagram of a solar cell, the SEM image of PEDOT:PSS on a Si substrate, and the integrated system's equivalent circuit. Reproduced with permission, from ref (Liu et al., 2018b), Copyright 2018, American Chemical Society. (F and G) The schematic diagram of the self-powered hydrogen generation system and the rate of hydrogen production at different conditions. Reproduced with permission, from ref (Wei et al., 2020), Copyright 2020, Springer.

Researchers proposed a solar and raindrops energy harvesting device (Liu et al., 2018b) by utilizing a poly(3,4-ethylenedioxythiophene):poly(styrenesulfonate) (PEDOT:PSS) layer as the mutual electrode of single-electrode mode TENG and solar cell. Figure 11A demonstrates the integrated hybridized energy harvester; the solar cell can generate higher output current density for PEDOT:PSS can reduce the light reflection (the band energy diagram of a solar cell in Figure 11D), and Figure 11C is the image of PEDOT:PSS on SEM Si substrate. Raindrops drive the operating principle of raindrops-based TENG as shown in Figure 11B, and the TENG could obtain an electric output of 33.0 nA/2.14 V. The equivalent-circuit diagram of the overall system is exhibited in Figure 11E; a rectifier was used to generate DC to charge a capacitor, and the hybridized energy collector generated a power density of 1.74 mW/m^2^ from the environment. In addition to the planar structure, the rotatory-disc-shaped devices are also commonly used in energy harvester for their unique structural characteristics. Lately, a hybridized energy system based on a rotatable TENG and WO_3_/BiVO_4_ heterojunction solar cell has been demonstrated, which can harvest mechanical energy in water (Wei et al., 2020). The schematic of the self-powered hydrogen generation system is exhibited in Figure 11F, and the insert is the photograph of the TENG model. The hybrid device of mechanical and solar energy could be utilized as a power supply of the water-splitting process; the hydrogen generation rate (water-splitting) is closely related to a rotational speed of TENG and light illumination on photoanode. The rate of hydrogen production under different conditions is shown in Figure 11G, indicating hydrogen production rate increases with the enhancement of rotation speed and light illumination, and the illumination could enhance the H_2_ generation rate from 5.45 μL min^−1^ to 7.27 μL min^−1^ under 160 rpm.

### Hybridized nanogenerators with solar-thermal-pyroelectric harvester

The solar-thermal-pyroelectric technology could scavenge the abundant renewable solar energy as the thermal source and convert it into electricity output directly based on the thermoelectric principle. The cost of preparation can be significantly diminished by solar-thermal-pyroelectric technology, thus avoiding the use of expensive silicon-crystal materials. What is more, this form of solar energy utilization has an advantage over other solar energy conversion forms like flexibility and portability. It can produce steady electricity even at night and continuously generate sustainable electricity, which has attracted researchers' significant attention.

**Figure 12:**
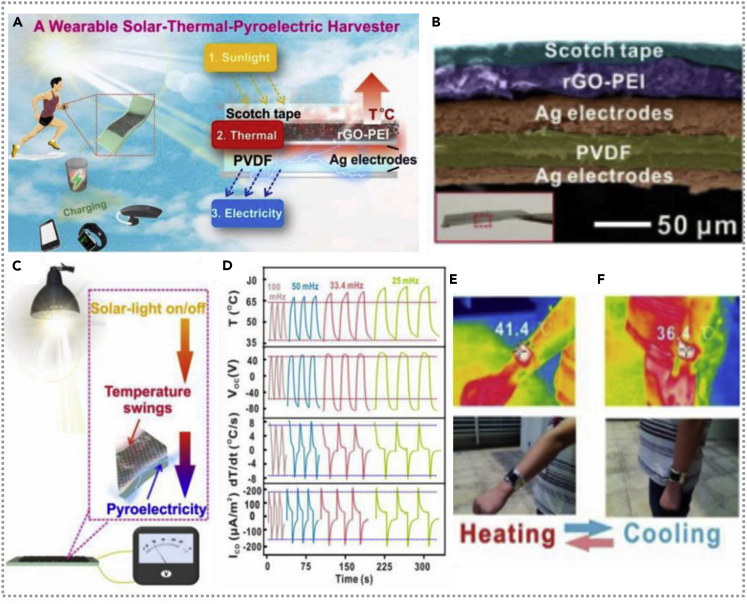
Hybridized nanogenerators with solar-thermal-pyroelectric harvester (A and B) The demonstration of mechanism of the energy-system-device-based solar-thermal pyroelectric harvester and detailed cross-sectional SEM image and photo of the prepared NG. Reproduced with permission, from ref (Li et al., 2020), Copyright 2020, Elsevier Ltd. (C and D) The demonstration of the sunlight-induced pyroelectric device from top to bottom and the short circuit current density of sunlight-triggered pyroelectric nanogenerator. Reproduced with permission, from ref (Li et al., 2020), Copyright 2020, Elsevier Ltd. (E and F) The surface temperature of the pyroelectricity device was measured in natural outdoor conditions during the hand-waving movements. Reproduced with permission, from ref (Li et al., 2020), Copyright 2020, Elsevier Ltd.

A sunlight-driven pyroelectric nanogenerator with efficient solar energy harvesting ability was fabricated (Li et al., 2020), the NG could be used as a comfortable wearable power supply during outdoor exercises, and the device (left) and working principle (right) are demonstrated. The pyroelectricity of the energy system is obtained as shown in Figure 12A. A detailed cross-sectional SEM image of the prepared NG and a cross-sectional digital picture for the ready device are exhibited in Figure 12B. The polyethyleneimine (PEI) was utilized to chemically modify reduced graphene oxide (rGO) and obtain an rGO-PEI film, which increased the π electron densities light absorbance by employing the amine functionalities of PEI on the rGO-PEI material. The dT/dt of rGO-PEI based device could be as high as 7.8 ± 0.2°C/s under sunlight. Figure 12C is the demonstration of the sunlight-induced pyroelectric device. Researchers measured the temperature distribution of the triggered pyroelectric nanogenerator under the light oscillation frequencies of 25, 33.4, 50, and 100 mHz, the corresponding open-circuit voltage (*V*_*OC*_), the temperature changing rate, and short-circuit current density (*I*_*CD*_). From top to bottom, the data chart is presented in Figure 12D. The fabricated pyroelectric nanogenerator was worn on the wrist as a bracelet, and the surface temperature of it was measured in the natural outdoor condition during the hand-waving movements (Figures 12E and 12F). The results indicated the temperature of the bracelet could change sharply during hand movement and generate a higher output current with hand moving.

## Application

**Figure 13:**
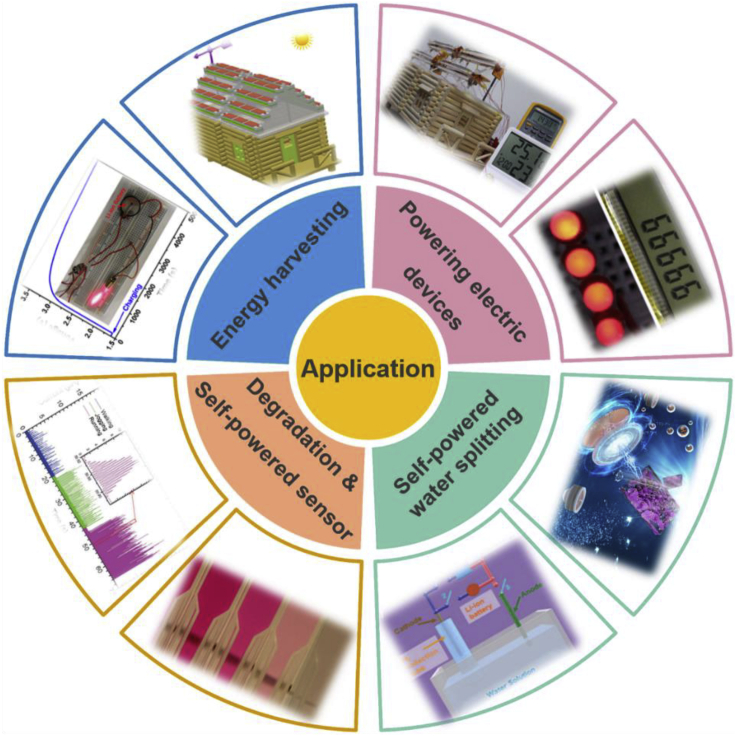
Typical applications of hybridized nanogenerators in different fields Clockwise from the first quadrant (powering electric devices). Reproduced with permission, from ref (Wang et al., 2016a), Copyright 2016, American Chemical Society. Reproduced with permission, from ref (Yang et al., 2013c), Copyright 2013, American Chemical Society. Reproduced with permission, from ref (Wei et al., 2020), Copyright 2020, Springer. Reproduced with permission, from ref (Yang et al., 2013a), Copyright 2013, Royal Society of Chemistry. Reproduced with permission, from ref (Yang et al., 2013b), Copyright 2013, American Chemical Society. Reproduced with permission, from ref (Pu et al., 2016), Copyright 2016, Wiley-VCH. Reproduced with permission, from ref (Yang et al., 2013b), Copyright 2013, American Chemical Society. Reproduced with permission, from ref (Wang et al., 2016a, 2016b, 2016c), Copyright 2016, American Chemical Society.

Hybridized nanogenerators could collect abundant solar and mechanical energy simultaneously or separately. After decades of exploration and development, a wide variety of materials and structures are employed to enhance and optimize renewable energy harvester's property gradually. Plenty of typical and significant workings suggest the great potential applications of hybridized nanogenerators in different fields, including energy harvesting system (Gao et al., 2020; Wu et al., 2014; Yang et al., 2013c; Zhao et al., 2017, 2020b), powering electric devices (Jiang et al., 2017; Quan and Yang, 2016b; Wang et al., 2016a; Yang et al., 2013c; Zhang et al., 2016b), self-powered water splitting component (Yang et al., 2013a), self-powered sensor (Qian and Jing, 2018; Wang et al., 2015a, 2016a; Wang, 2014; Wen et al., 2016; Zhang et al., 2014; Zhao et al., 2019), and self-powered electron degradation units (Wei et al., 2020; Yang et al., 2013b). These versatile applications have epoch-making significance for the era of artificial intelligence and new energy, and some demonstrations are exhibited in Figure 13.

All of the various kinds of hybridized nanogenerators could scavenge solar and mechanical energy ubiquitously in the environments and generate electrical output based on the photoelectric effect, friction electrification-electrostatic induction, piezoelectric effect, etc. Table 1 in this paper depicts the electrical characteristics of hybridized nanogenerators utilized as energy harvesting systems, including the whole output performance of hybridized nanogenerators, the type of solar/mechanical energy harvesters, and the electrical property of solar/mechanical energy collecting. For example, Yang et al. constructed an efficient hybridized nanogenerator (Wang et al., 2016a) that consisted of commercial solar cells and wind-driven TENG. Under a full-sun intensity and 15 m/s, the single solar cell and TENG could generate output signals of 9 mA/7 V/8 mW and 260 μA/26 mW, respectively. The hybridized nanogenerator could be integrated into parallelly and installed on the roofs of buildings, which maximize the use of wind and solar energy without negative impact on people's production and daily life.

**Table 1:** A summary of the electrical characteristics of hybridized nanogenerators utilized as an energy harvesting system

Types of mechanical energy harvester	Electrical characteristics of mechanical energy harvester	Types of solar energy harvester	Electrical characteristics of solar energy harvester	Electrical characteristics of hybridized nanogenerator	References
Rain-TENG & Wind-TENG	5 V & 50 V 6 μA	Commercial solar cell	4.2 V, 27 μA		Roh et al. (2020)
Wind-TENG	231 nW	Phototransistor	15.4 μW		Zhang et al., 2016a
Wave-TENG & Wave-EMG	142 V, 23.3 μA, & 0.66 V 2.14 mA	Commercial solar cell	1.8 V 6.15 mA/cm^2^		Shao et al. (2017)
Waterdrops-TENG	33.0 nA, 2.14 V	Heterojunction silicon solar cell	0.612 V 29.4 mA/cm^2^		Liu et al., 2018b
Waterdrops-TENG	7 V, 128 nA 0.265 mW	Conventional solar cell			Jeon et al. (2015)
Wind-TENG		Dye-sensitized solar cell	0.7 V 14.4 mA	150 μA 2 mW	Cao et al. (2018)
Rotatory-shaped TENG	230 V 0.12 mA	Heterojunction photoanode	5.24 mA/cm^2^		Wei et al., 2020
TENG-PENG	3.8 μA, 44 nW	Photovoltaic cell	890 nA, 60 V 7 μW	5 μA 56 V	Zhang et al., 2017
Water-TENG & Wind-TENG	86 mW/m^2^ & 8 mW/m^2^	Commercial solar cell	0.6 V		Zheng et al. (2015)
Wind-TENG	260 μA, 26 mW	Si-based solar cell	9 mA, 7 V 8 mW	12 mA	Wang et al., 2016a
Human biomechanical energy-TENG	0.95 mA/cm^2^ 1.25 V	Organic solar cell	5.8 mA/cm^2^ 0.74 V	1.9 V	Fang et al. (2015)
PENG		Heterojunction solar cell	0.41 V 31 μA/cm^2^		Yang et al. (2013c)
Contact/separation TENG	110 V, 60 μA	Si-based solar cell	0.62 V 32 mA/cm^2^	3.5 V 30 mA	Yang et al. (2013a)
Contact/separation TENG	18.2 V, 1.4 μA	Dye-sensitized solar cell	0.7 V	3.1 mA	Dudem et al. (2016)
Contact/separation TENG	3 V	Si pyramid solar cell	0.6V, 18mA 35 mA/cm^2^	12 V 17.4 mA	Yang et al. (2013b)
PENG	0.378 V 22.06 mA/cm^2^	Organic photovoltaic device	10.17 mA/cm^2^ 0.59 V	0. 71 V	Yoon et al. (2015)

Hybridized nanogenerators could generate steady and continuous electrical output with considerable power to drive multiple electric devices, a promising application for the artificial intelligence era and the IoT. Yang and co-workers have fabricated a flexible hybridized energy harvester consisting of ZnO-P3HT heterojunction solar cell and polarized PVDF film-based nanogenerator (Wang et al., 2016a). Solar, mechanical, and thermal energy could be scavenged simultaneously or separately, and LCD can be actuated by hand-touching even more; the power could not only be stored in Li-battery but also light four LEDs. The demonstration is as shown in the inset upper right corner of Figure 13.

Yang et al. fabricated a novel hybridized energy cell (Yang et al., 2013b) that could be directly utilized as a self-powered electron degradation of rhodamine B(RhB) with superior degradation percentage in a short time. The hybridized energy cell consists of a TENG and solar cell, scavenging mechanical and solar energy simultaneously and delivering excellent electricity. The electricity could not only be stored in a Li-ion battery but also be employed for electron degradation. The color change of the RhB solution under different electron degradation times is shown in Figure 13, and the degradation percentage of RhB is as high as 98% in 10 min.

## Summary, prospective, and future challenges

We reviewed the development of hybridized nanogenerators, including the importance of multi-type energy harvesting and the working mechanism of solar and mechanical energy scavenging. Moreover, mechanical harvesting energy and solar energy nanogenerator classification is also discussed according to mechanical energy and solar cell types, respectively. The diverse applications of hybridized nanogenerators are being reviewed in detail. Finally, the challenges and prospects of hybridized nanogenerators and the future explored improvement in output performance, stability, preparation, large-scale utilizing, and efficiency will be discussed.

The structural design and property enhancement of hybridized nanogenerators had been studied widely and developed rapidly in the past few decades. However, the overwhelming majority of the hybridized energy cell is restricted to the laboratory's theoretical research and experimental stage. Beyond that, it is a formidable goal to construct a large-scale, low-cost fabrication, and the research cycle of researchers is still very long. How to effectively enhance the energy harvesting properties of materials, collect solar energy, make the hybridized nanogenerator portable and lightweight, scavenge, and convert energy efficiently is a very practical and critical problem ensuring no apparent attenuation in energy harvester performance and considerable output power. It is also a problem that hybrid nanogenerators are expected to apply. Researchers still need to devote themselves to the material and construction of hybridized nanogenerators and make this energy technology universal in energy and artificial intelligence worldwide.

**Figure 14:**
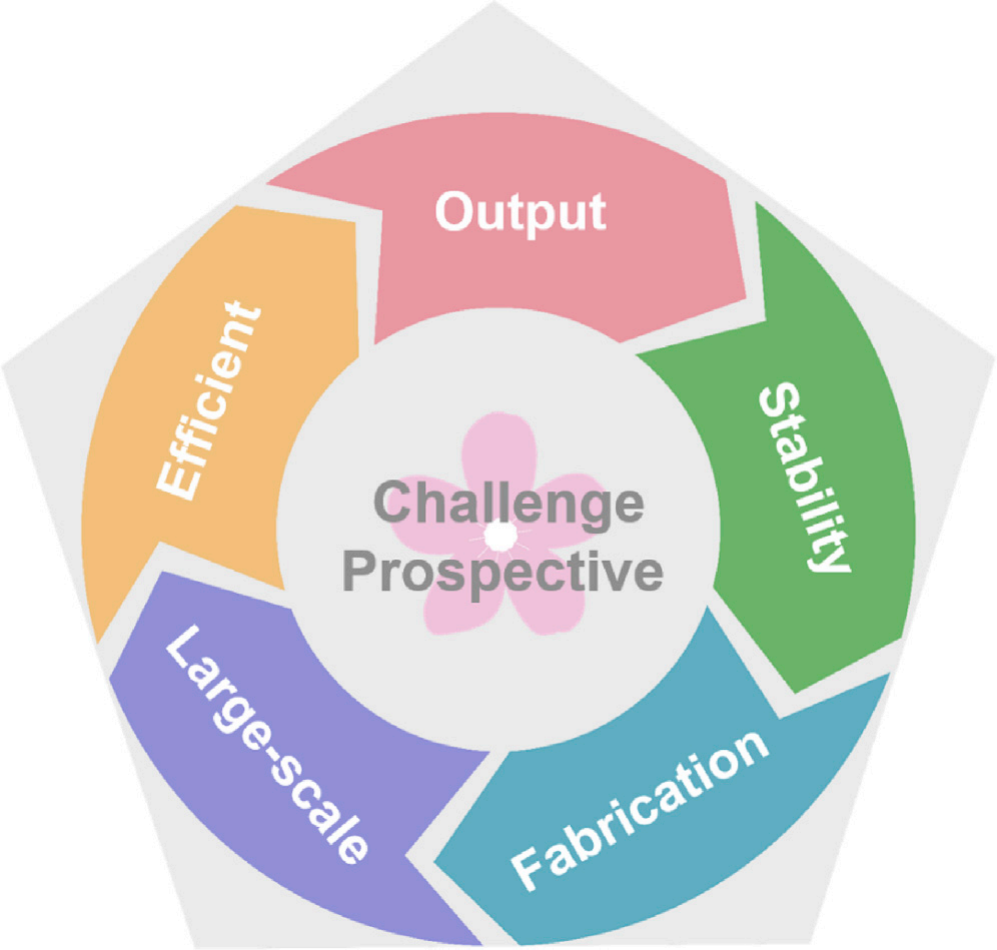
The challenges and perspectives of hybridized nanogenerators for scavenging mechanical and solar energies.
